# Is HIV-1 RNA dimerization a prerequisite for packaging? Yes, no, probably?

**DOI:** 10.1186/1742-4690-1-23

**Published:** 2004-09-02

**Authors:** Rodney S Russell, Chen Liang, Mark A Wainberg

**Affiliations:** 1 McGill AIDS Centre, Lady Davis Institute, Jewish General Hospital, 3755 Cote Ste-Catherine Road Montreal, Quebec, Canada H3T 1E2; 2 Department of Microbiology & Immunology Montreal, Quebec, Canada H3A 2B4; 3 Department of Medicine, McGill University, Montreal, Quebec, Canada H3A 2B4

## Abstract

During virus assembly, all retroviruses specifically encapsidate two copies of full-length viral genomic RNA in the form of a non-covalently linked RNA dimer. The absolute conservation of this unique genome structure within the *Retroviridae *family is strong evidence that a dimerized genome is of critical importance to the viral life cycle. An obvious hypothesis is that retroviruses have evolved to preferentially package two copies of genomic RNA, and that dimerization ensures the proper packaging specificity for such a genome. However, this implies that dimerization must be a prerequisite for genome encapsidation, a notion that has been debated for many years. In this article, we review retroviral RNA dimerization and packaging, highlighting the research that has attempted to dissect the intricate relationship between these two processes in the context of HIV-1, and discuss the therapeutic potential of these putative antiretroviral targets.

## Introduction

The dimeric feature of the retroviral RNA genome was identified almost forty years ago. However, as with many topics in retrovirology, interest in this area was heightened with the realization that the causative agent of AIDS was a retrovirus. Since then, RNA and protein sequences involved in genome dimerization have been identified for a number of retroviruses, and the dimeric nature of the retroviral genome is known to be important for various critical events in the viral life cycle. These include reverse transcription and recombination, as well as genome encapsidation. To date, a number of informative reviews have been published on retroviral RNA dimerization [1-3], genome packaging [3-7], and the role of nucleocapsid (NC) protein in these activities [8,9]. More recently, a comprehensive review was published that summarized the contributions of *in vitro *analysis to the identification of retroviral dimerization signals, and provided an overview of the HIV-1 5' untranslated region (UTR) structure with reference to a number of proposed models [10]. Another, in this issue of *Retrovirology*, focuses on the different roles of different dimer linkage structures amongst various retroviruses [11]. In this review, we will focus on results from *in vivo *studies that provide insights into the relationship between retroviral RNA dimerization and packaging, and the biological relevance of these activities to viral replication.

## Retroviral RNA dimerization

The first evidence for the existence of a dimerized RNA genome came in 1967 when it was shown that viral RNA from each of Rous sarcoma virus (RSV), avian myeloblastosis virus (AMV), murine leukemia virus (MLV), and mouse mammary tumor virus (MTV) displayed sedimentation constants between 64S and 74S in sucrose gradients [12]. Since these sedimentation constants and corresponding molecular weights were much larger than those of most other known viral RNAs, the structure of these RNA genomes became a matter of great interest. Experiments showing that the 62S RSV RNA species could be converted to a 36S species by heat treatment suggested a disaggregation of the 62S RNA into smaller RNAs, and implied that the fast-sedimenting (62S) RSV RNA was actually an aggregate of smaller (36S) RNAs [13]. The first real understanding of this putative aggregate RNA structure came in 1975 when RNA from the endogenous feline retrovirus, RD-114, was visualized by electron microscopy (EM), and it was apparent that the 52S RNA molecule existed as an extended single strand that contained a central Y- or T-shaped secondary structure [14]. It appeared that this 52S molecule actually consisted of two half-size molecules, joined together by the Y- or T-shaped structure, which was termed rabbit ears (RE). It was later shown that the RNA had a poly(A) sequence at each of the two free ends. More importantly, this indicated that nucleotides involved in this RE, or dimer linkage structure (DLS), resided in the 5' region of the RNA genome [15]. Similar structures were also reported for numerous other type C RNA viruses [16-21]. The absolute conservation of a DLS among retroviruses was strong evidence that the dimerization process must be critical to the retroviral life cycle. With the discovery that the causative agent of AIDS was also a retrovirus, inhibition of RNA dimerization was proposed as a possible therapy for HIV, and HIV-1 RNA dimerization became an intensely studied topic.

Both *in vivo *and *in vitro *approaches have been used to study retroviral RNA dimerization. The *in vivo *approach is that whereby RNA is isolated from virions produced in tissue culture and then analyzed by native Northern blotting [22]. The other method involves synthesis of short segments of viral RNA *in vitro*, and then studying the ability of these fragments to form dimers. The HIV-1 DLS was originally identified when it was shown that an *in vitro*-transcribed fragment of HIV-1 RNA could form two major bands on a native gel after incubation at 37°C for 15 min [23]. The lower band had the expected size of the RNA fragment, while the upper band corresponded to a dimer. *In vivo *evidence for a role of the NC protein in the dimerization process was already available [24], and this study also showed that NC could bind to viral RNA and increase the rate of dimerization of the RNA fragments in these *in vitro *dimerization assays [25].

**Figure 1:**
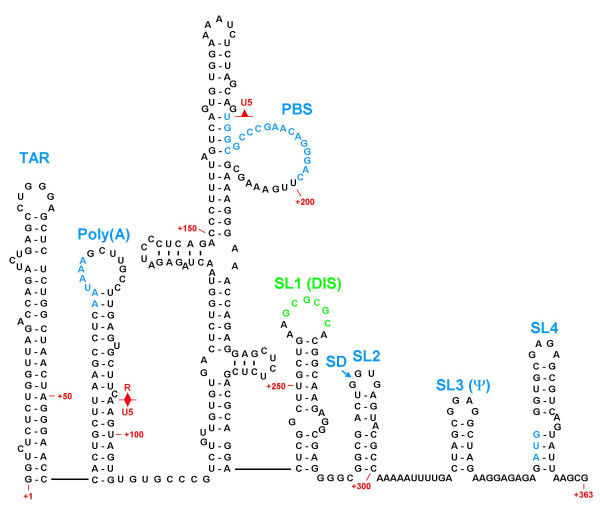
**HIV-1 5' RNA Structural Elements. **Illustration of a working model of the HIV-1 5' UTR showing the various stem-loop structures important for virus replication. These are the TAR element, the poly(A) hairpin, the U5-PBS complex, and stem-loops 1–4 containing the DIS, the major splice donor, the major packaging signal, and the *gag *start codon, respectively. Nucleotides and numbering correspond to the HIV-1 HXB2 sequence. (Adapted from Clever *et al*. [73] and Berkhout and van Wamel [136])

It was subsequently reported that an RNA fragment representing nt 1–311 of HIV-1 RNA (Mal strain; a chimera of subtypes A and D) could not only form dimers, but that RNAs containing these first 311 nt could dimerize 10 times faster than RNA sequences at positions 311–415 that were previously shown to be sufficient for HIV-1 RNA dimerization [25]. Based on these results, the authors concluded that sequences upstream of the splice donor site are involved in the dimerization process, and proposed that sequences in this region somehow hastened the reaction. The key nucleotides involved in this RNA dimerization event make up a palindromic sequence, 274-GUGCAC-279, between the PBS and the major splice donor [26], and RNA sequences on both sides of this palindrome can form a stem-loop structure with the palindrome in the hairpin loop. Deletion of this stem-loop motif (nt 265–287) completely abolished dimerization of the 1–615 HIV-1 RNA fragment *in vitro*. The palindromic region was termed the dimerization initiation site (DIS) and it was proposed that this structural element could be exploited for targeted antiviral therapy by antisense oligonucleotides [26]. These findings were later confirmed when a 19 nt sequence upstream of the 5' major SD was shown to be part of the HIV-1 RNA dimerization domain (Lai strain; subtype B) [27], and it was found that *in vitro *dimerization of a 224–402 nt RNA fragment was completely blocked by an antisense oligonucleotide that targeted the palindrome [28]. This led to a "loop-loop kissing complex" [29] or "kissing-loop model" [27] of HIV-1 RNA dimerization, in which the 6 nt palindromes on each of the two monomeric RNA molecules interact through Watson-Crick base-pairing. Purine residues flanking the palindrome were later shown to be intricately involved in this initial interaction [30,31] which is believed to shift the equilibrium toward the formation of dimers, allowing the stems to melt and anneal to their complementary sequences on the other RNA molecule, thus forming the stable extended duplex (Fig. 1). This model fits with the idea that immature virions contain a less stable dimer involving only base-pairing of the palindromes, but that the mature virions contain a more stable structure, the extended duplex. Subsequent phylogenetic analysis of over 50 HIV-1, HIV-2, and simian immunodeficiency virus (SIV) nucleotide sequences showed an absolute conservation of a predictable structure similar to the DIS, with the hallmark of the HIV-1 DIS motif being a 6 nt palindrome consisting of either a GCGCGC or a GUGCAC sequence [32,33]. Similar kissing-loop models have also been proposed for a number of other retroviruses [34-41].

Despite ample *in vitro *evidence supporting the above model of dimer maturation, it was not yet known where or when the RNA dimer was actually formed *in vivo*. However, native Northern blotting analysis of RNA from two Moloney murine leukemia virus (MuLV) protease-negative (PR^-^) mutants displayed dimers that migrated more slowly, and showed lower melting temperatures, than that of wild-type [42]. It was therefore concluded that PR function is required for RNA maturation in MuLV. Similar experiments with a related virus also suggested that the RNA maturation event required an intact, unsubstituted Cys array within the NC domain [42]. On the basis of these results, a maturation pathway was proposed for MuLV in which Gag polyprotein molecules assemble into a nascent virion containing an immature dimer. The particle would then be released from the cell, and once Gag is cleaved by PR, NC would act on the immature dimer, converting it to the mature form.

Evidence for the role of NC in this dimer maturation process came when *in vitro *analysis showed that NC could convert the less thermostable dimers to a more stable conformation [43]. Similar results were obtained by others showing that HIV-1 NC could activate dimerization of a 77–402 nt fragment of HIV-1 Lai RNA, as well as convert an unstable dimer, corresponding to the kissing complex, to a stable one [44]. Taken together, these thermostability conversions seem to resemble the RNA maturations reported *in vivo*, and, in agreement with earlier proposals [24], strongly suggest that NC is responsible for the dimer maturation depicted in Fig. 1. Subsequent *in vivo *analysis of a panel of HIV-1 NC mutants showed that Cys-Ser substitution of amino acid residues within the second zinc finger decreased genomic RNA dimerization to the same extent as disruption of the DIS [45]. This finding confirmed the involvement of NC in the dimerization process, and suggests that the kissing-loop model also applies to the *in vivo *situation.

## HIV-1 RNA packaging

Why a class of viruses would evolve to have such a unique genomic structure is not entirely clear, but it is speculated that the availability of two copies of the genome would be advantageous for recombination during the complex reverse transcription process, that is key to the retroviral life cycle [46]. Indeed, the dimeric nature of the genome is thought to be responsible for a high rate of recombination during infection [47-50]. Given that most dimerization signals overlap with known packaging elements, it was naturally assumed that it is the RNA dimer that is specifically recognized for packaging in the case of retroviruses, and that this dimeric feature ensures proper packaging of two copies of genomic RNA. A number of studies have attempted to address this question of a link between dimerization and packaging, but let us first review several aspects of the HIV-1 RNA packaging process.

**Figure 2:**
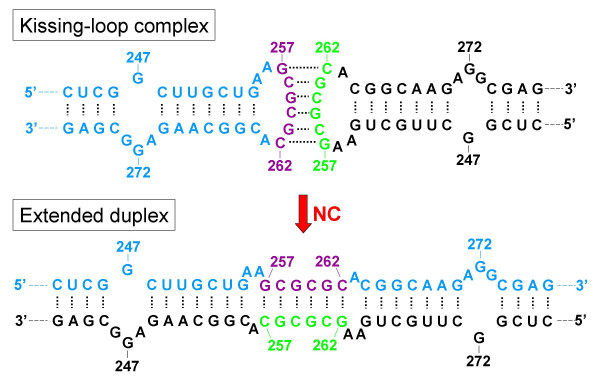
**The Kissing-Loop Model of HIV-1 RNA Dimerization. **HIV-1 RNA dimerization is initiated by a Watson-Crick base-pairing interaction between two palindromes in the loops of SL1 on two monomeric genomic RNAs. This interaction forms the loose unstable kissing-loop complex. Coincident with virus particle maturation, this unstable dimer is rearranged to form a more stable extended duplex that involves a mechanism whereby the base-pairs in the stems melt and then re-anneal to their complementary sequences on the opposite strand. Nucleotides and numbering correspond to the HIV-1 HXB2 sequence. (Adapted from Skripkin *et al. *[26] and Laughrea and Jetté [27])

The first studies aimed at identifying the HIV-1 RNA packaging signal found that deletion of RNA sequences between the major splice donor (SD) and the *gag *coding region (i.e. SL3 and adjacent sequences in Fig. 2) decreased the levels of genomic RNA packaged into virions [51-53]. Since these sequences were downstream of the major 5' SD, and therefore would not be found in any spliced viral RNA species, it was plausible that this region could be responsible for the selective packaging of genomic RNAs. Analysis of the putative ψ locus from a variety of retroviruses showed that these sequences had the ability to direct the selective encapsidation of heterologous RNAs to which they had been linked artificially [54-61]. In HIV-1, such autonomous packaging signals were mapped to the regions extending 30–40 nt immediately upstream and downstream of the *gag *start codon [62]; however, subsequent studies showed that RNA sequences upstream of the 5' SD site also affected RNA packaging [63]. It was also known that retroviral encapsidation required trans-acting amino acid sequences in the Gag protein [51,64-68], and several groups reported that HIV-1 Gag and NC exhibit specific binding affinity for the HIV-1 ψ site *in vitro *[23,69-72]. These findings, combined with chemical and RNase accessibility mapping, as well as computerized sequence analysis, led to the generation of a model for the HIV-1 ψ site that comprised four independent stem-loops [73] (SL1-4 in Fig. 2). Three of these hypothetical stem-loop structures were each shown to serve as independent Gag binding sites, and were proposed to contribute individually to overall packaging efficiency. SL1, SL3, and SL4 were later shown to be critical for packaging specificity *in vivo *[74,75]. Subsequent *in vitro *analysis from another group demonstrated that the major packaging signal is an extended bulged stem-loop whose RNA conformation is altered upon interaction with Gag [76]. However, more recent work indicates that SL2 and SL3 display much higher affinities for NC than SL1 and SL4 *in vitro *[77,78]. Based on these findings, a model has been proposed to represent the initial complex formed between the NC domains of assembling Gag molecules and the dimeric ψ region [79]. In this model, SL1 is shown to form an RNA duplex between the two stands, while SL4, instead of directly binding to Gag, contributes additional RNA-RNA interactions that stabilize the tertiary structure of the ψ element. The RNA conformation resulting from this folding pattern is thought to expose SL2 and SL3 for high-affinity binding to Gag.

Despite the clear results obtained from simplified *in vitro *studies such as those mentioned above, the SL1-4 region alone is not sufficient to target RNA into HIV-1 virions *in vivo *[80], and the minimal region required to confer autonomous packaging activity actually maps to a larger region covering the first 350–400 nt of the genome, including ≈ 240 nt upstream of SL1 [81-84]. In agreement with these studies, mutations that alter the stability of the poly(A) hairpin stem region, or delete the upper part of the hairpin, severely inhibited HIV-1 replication [85]. And, these deficits in replication correlated with reduced RNA packaging levels in virions, suggesting that the formation of the poly(A) hairpin is necessary for normal packaging of viral genomes. Subsequent research confirmed the importance of the poly(A) hairpin in the RNA packaging process [86], and it was shown that similar disruption of base-pairing in the stem of the TAR element also caused profound defects in packaging [81,86]. Finally, deletion analyses of RNA sequences between the poly(A) hairpin and SL1 suggested that unspecified sequences within the U5-PBS region also contribute to HIV-1 RNA packaging [83,86]. Our group later showed that GU-rich sequences in the lower stems of the poly(A) hairpin and the U5-PBS complex contribute to both dimerization and packaging [87].

In summary, all of the seven predicted stem-loop structures in the HIV-1 5' UTR (Fig. 2) are known to be important for genome encapsidation, and all of these RNA structural elements have also been assigned other functions in various steps of the viral life cycle, e.g. the role of SL1 in the initiation of dimerization. The existence of such overlapping functions for these RNA structures raises the possibility that some of these functions, such as dimerization and packaging, might be linked. The evidence for and against the existence of such a link in HIV-1 will be the main focus of the remainder of this review.

## Is dimerization a prerequisite for packaging?

One of the first electron microscopy studies of a retroviral DLS in 1976 proposed that this region "could have some role in packaging the RNA in the virus" [16]. This raised the question of a possible link between dimerization and packaging that is still debated. The answer to this question has significance in our basic understanding of the retroviral life cycle and may also have implications for therapy, since many groups are actively studying these two activities as potential drug targets.

### Clues from *in vitro *studies

Early reports on *in vitro *dimerization of HIV-1 RNA showed that the DLS localized to a stretch of genomic RNA downstream of the 5' SD (nt 311–415) [23,88], and it was noted that this dimerization domain encompassed a previously identified packaging element that had also been shown to bind NC [51-53]. This dependence of HIV-1 RNA dimerization on *cis *elements required for packaging was immediately interpreted to mean that retroviral RNA dimerization, activated by either NC or Gag precursors, should direct genomic RNA into the virion, implying that dimerization might be a prerequisite for packaging. Since HIV-1, MuLV, and RSV all contain elements involved in dimerization that were also required for packaging [23,89,90], it was proposed that dimerization might function as a molecular switch that negatively regulates translation and positively regulates encapsidation [88]. The existence of a DLS downstream of the major splice donor would seemingly supply a convenient mechanism whereby only genome length RNA would be able to dimerize and subsequently become encapsidated into the virion. However, evidence questioning such a dimerization-mediated mechanism of genomic RNA packaging came from studies showing that sequences upstream of the SD site had even greater dimerization capabilities than those located downstream [25-27]. The involvement of such sequences (e.g. the DIS, SL1) in the dimerization process questioned the link between dimerization and packaging, because these sequences are also found in all HIV-1 spliced viral RNAs.

### Observations from *in vivo *studies

Early *in vivo *studies analyzing the structure of virion-associated RNA from rapid-harvest avian retroviruses showed that viral RNA appeared to be a mixture of monomers and dimers [91-93]. Similar results had also been reported with PR [94,95] and NC [24,94,96,97] mutants, which argued against the notion that dimerization is a prerequisite for packaging. However, analysis of rapid-harvest virus in MuLV showed that genomic RNA was already in the form of a dimer shortly after budding, albeit as a less stable, physically different RNA dimer than that present in mature virions [42]. Based on these observations it was proposed that MuLV particles never package monomeric RNAs, but rather that the dimeric RNA structure might be integral to the packaging signal that is recognized by Gag during assembly. It was also speculated that the previously reported presence of monomers in viral RNA preparations had resulted from the physical dissociation of fragile unstable dimers during RNA preparation. Similar experiments performed on PR^- ^mutants of HIV-1 showed that substantial amounts of monomeric RNA could be detected [98]. Since PR^- ^dimers were shown to be less stable than wild-type dimers, it was assumed that dimers were preferentially packaged in PR^- ^particles, but that some fragile dimeric structures had dissociated during RNA preparation. Based on these *in vivo *results with both MuLV and HIV-1, it was concluded that dimerization is a prerequisite for packaging and should be considered to be a general feature of retrovirus assembly.

Further insights into this topic can be obtained by examination of results from a number of studies aimed at understanding the role of the DIS in HIV-1 replication. One such study, in which DIS loop palindrome sequences were mutated, found that mutation of the palindrome to shorter or longer versions of GC stretches did not have major effects on viral RNA dimerization; however, partial RNA packaging defects were observed that also corresponded to diminutions in viral replication [33]. Based on these data, it was proposed that these DIS loop mutants might have experienced a partial dimerization defect that caused inefficient packaging [33]. In a similar study, mutation of the palindrome, as well as deletion of the upper stem-loop of SL1 caused drastic reductions in viral infectivity and decreases in both dimerization and packaging of HIV-1 genomic RNA [32]. In an attempt to explain how these mutations could affect both activities, a model was proposed in which Gag does not specifically recognize the dimerized genome but rather initially interacts with one molecule of genomic RNA that happens to be linked (dimerized) to a second such molecule. Then, during packaging, Gag would effectively bind to two genomic RNA molecules at once. Hence, defects in dimerization would result in subsequent packaging defects. Based on these data, it was also concluded that the encapsidation and dimerization processes are coupled to some extent.

Although several groups had attempted to delineate the relationship between dimerization and packaging, the fact remains that the RNA signals that are important for both of these activities overlap in most retroviral genomes; this makes it difficult to interpret the results of mutagenesis studies. In an attempt to generate viruses that would be expected to display selective defects in dimerization or packaging, one group designed a panel of constructs containing mutations in SL1, SL3, or both [99]. Results from this study showed that deletion of either SL1 alone, or SL3 plus adjacent flanking sequences, reduced genomic packaging, while deletion of SL1 and SL3 simultaneously caused an even further reduction. With respect to dimerization, complete deletion of SL1, or even disruption of the base-pairing in the upper stem, resulted in elevated levels of monomer-sized RNA species on native Northern blots, again confirming the importance of this region for the *in vivo *HIV-1 RNA dimerization process. Yet, these mutant genomes could still be packaged, suggesting that HIV-1 RNAs need not be dimers for this to happen. Thus, the authors concluded that dimerization is not a prerequisite for packaging but rather serves an independent function in the retroviral life cycle. In the above-cited article, the effects of SL3 mutations on dimerization were not studied, but our group later showed that viruses containing even minor substitutions in or around SL3 could have significant effects on both dimerization and packaging [100,101].

In summary, the *in vivo *studies described above commonly observed that mutations in 5' RNA sequences affected both dimerization and packaging, presumably due to the close proximity of the RNA dimerization and packaging signals.

### Can monomers be packaged?

In an attempt to separate the dimerization and packaging functions, and to characterize the DIS-DLS region without altering packaging activity, one group generated mutant constructs carrying a duplication of approximately 1000 nt from the HIV-1 5' region (termed E/DLS) including the encapsidation signal and the DIS-DLS [102]. They found that the presence of an ectopic E/DLS near the 3' region of the genome resulted in the appearance of monomeric RNA in virus particles, suggesting that monomers can be packaged and that dimerization of HIV-1 genomic RNA is not required for packaging. However, they also found that two intact E/DLS regions had to be present on the same RNA molecule in order for packaging of monomers to occur. Therefore, it was assumed that these monomers had been generated from an intramolecular interaction between the two E/DLS regions. If we assume that such an intramolecular interaction between two DLS structures would occur on a single RNA molecule, however, might such a structure then not also appear as a dimer to a Gag protein that was attempting to package it? Although these data were interpreted to mean that dimerization is not required for packaging, they also suggest that some structure that is generated by the interaction of the two E/DLS regions might be recognized by Gag in order to facilitate packaging. In the context of wild-type genomic RNA containing only one E/DLS region, such a structure might then only be generated by an intermolecular interaction between two RNA molecules, i.e. a dimer. Hence, these results also imply that dimerization might be required for proper packaging.

In a follow-up study, the same group created mutant HIV-1 particles that contained only monomeric RNAs, and concluded that these mutants demonstrated the complete separation of encapsidation from physical dimerization of retroviral RNA [103]. However, they also reported that these viruses packaged only monomers, and that packaging efficiencies were approximately half those of wild-type, implying that dimerization is the sole mechanism to ensure the packaging of two copies of viral genomic RNA into each virus particle. In addition, the packaged monomers might have originally been weak dimers that dissociated during extraction and analysis, as has been pointed out in previous reports [42,102].

However, the above results do raise the issue of packaging specificity in mutant viruses. We and others have shown that, in COS cells, HIV-1 can incorporate significant amounts of spliced viral RNA when proper packaging of full-length viral genomic RNA is reduced [99,104]. During assembly, Gag will always successfully package some RNA, and it is important to know the degree of specificity with which monomers versus dimers are packaged. If monomer-packaging mutants concomitantly package high levels of spliced viral RNA, then it is likely that packaging specificity may have been compromised by the existence of an extra E/DLS, and that the packaging of the monomers was non-specific. However, a lack of spliced viral RNA in these virions would indicate that the monomers were packaged with a high degree of specificity, and would have implications as to whether or not Gag initially recognizes viral genomic RNA in a dimeric versus a monomeric state. None of the viruses engineered to package only monomers were able to efficiently establish a new round of infection, suggesting that dimerization is required for replication if not for packaging. It is difficult to predict what other effects the addition of large segments of highly structured RNA might have on the viral life cycle.

Another group reported similar phenotypes in the context of an HIV-1 mutant that was designed to have altered Gag/Gag-Pol ratios [105]. Analysis of virion-derived genomic RNA from these viruses showed an increase in packaging of monomers, demonstrating that stable RNA dimers are not required for encapsidation of HIV-1 genomic RNA. Interestingly, these viruses also showed drastically reduced infectivity.

### Insights from forced evolution studies

We have also been studying the HIV-1 5' UTR and its putative interactions with Gag, and how these interactions affect dimerization and packaging activities. The DIS is known to be important for viral replication [32,33,63,99,106-109], reverse transcription [47,48,107,109], RNA dimerization [32,99,106,109-111], and packaging [32,33,74,99,107,108,110], as well as packaging specificity [99]. However, despite the obvious importance of this stem-loop structure, work from our group has shown that defective viral replication caused by deletions in the DIS can be largely corrected by a series of compensatory point mutations identified in matrix, capsid, p2, and NC [112-114]. These findings imply that the RNA sequences comprising the DIS interact in some way with these domains of Gag, and that when the RNA sequences are mutated, the virus will acquire adaptive mutations that potentially restore putative RNA-protein interactions over long-term culture. Since the originally deleted RNA sequences were in the DIS, we had naturally assumed that the major defect of these mutants would relate to RNA dimerization, and that compensatory mutations had arisen to correct defective RNA dimerization activity. To our surprise, this was not the case. Although our mutants did indeed yield reduced levels of dimerized genomic RNA in virus particles, the compensatory mutations in Gag that restored replication capacity [112-114] did not correct dimerization defects [109]. Rather, compensatory mutations apparently resulted in increased overall levels of viral genomic RNA that were packaged into virus particles, irrespective of impaired RNA dimerization. Similar effects on packaging were observed in the context of compensatory mutations identified during long-term culture of viruses containing mutations outside the DIS, such as the poly(A) hairpin and the U5-PBS complex [87], and between the PBS and SL1 [115]. These findings again question the link between dimerization and packaging, since our compensatory point mutations were able to increase RNA packaging levels without correcting dimerization. One possibility is that the revertant viruses somehow gained the ability to package wild-type levels of RNA without correcting dimerization defects, i.e. they packaged more monomers. However, we also cannot rule out the possibility that our point mutations in Gag may have restored weak dimerization properties to the mutated RNAs, and that the latter dimers dissociated during extraction and analysis.

In a follow-up study, we created two other DIS deletions and combined them with various combinations of the previously identified compensatory point mutations. We showed that these mutant viruses, ΔLoop (lacking the loop region of SL1) and ΔDIS (lacking the complete SL1) displayed defects in replication, RNA dimerization, and packaging. Once more, all of these but dimerization were largely corrected by the compensatory point mutations in Gag [104]. Even a virus that lacked the DIS, e.g. ΔDIS, and which never showed any signs of viral growth in tissue culture, was able to replicate to significant extent when it also possessed the compensatory mutations.

The mechanism(s) whereby these compensatory point mutations functioned to restore replication had eluded us for some time. Recently, however, we employed an RNase protection assay to discriminate between genomic and spliced viral RNA packaged into virus particles. Our results showed that all of our 5' UTR mutant viruses aberrantly packaged increased levels of spliced viral RNA compared to wild-type virions. More importantly, however, the effect of one of our compensatory point mutations (i.e. MP2; a Thr->Ile substitution at position 12 of the SP1 spacer peptide in Gag) was to exclude spliced viral RNA from being packaged into mutant virions [104]. Surprisingly, this single point mutation was also able to restore significant levels of virus replication to our ΔDIS mutant virus, which had been noninfectious in both T cell lines and blood mononuclear cells.

Previous work had suggested that the packaging of spliced viral RNA is a mechanism used by packaging mutants to fill the space that would normally be occupied by genomic RNA [99]. Were this the case, then the MP2-mediated exclusion of spliced viral RNA from the virus particle should have been accompanied by increased packaging of genomic RNA. In the absence of MP2, the mutant particles contained lower levels of genomic RNA and higher levels of spliced viral RNA packaged than wild-type. In contrast, the presence of MP2 led to the exclusion of spliced viral RNA, but had no effect on packaging of genomic RNA. In the context of dimerization and packaging in the mutated viruses, it is possible that spliced viral RNAs, which do contain some RNA elements involved in RNA dimerization, including the DIS, might form heterodimers with molecules of genomic RNA. These putative heterodimers might be packageable, but it is unlikely that virions containing such genomes would be able to replicate, e.g. the noninfectious ΔDIS mutant. However, in the presence of MP2, the modified Gag protein might in some way block the formation of such an RNA heterodimer, thereby increasing the probability that dimers form between two genomic RNA molecules, resulting in partially restored levels of virus replication. Since these genomic RNA molecules are already mutated in dimerization signals, these weaker dimers would probably appear on a gel as monomers. In such a model, MP2 would act to restore dimerization, resulting in increased replication capacity, suggesting that dimerization is required for proper packaging to ensure that a particle is infectious. Unfortunately, this is virtually impossible to prove with current *in vitro *and *in vivo *protocols. New approaches to study dimerization and packaging within the cell will hopefully allow new hypotheses to be tested.

The packaging of spliced viral RNA and/or the exclusion of such RNA species raises the question of whether the viral RNA sequence, or possibly the RNA structure, is important in proper assembly and/or structural integrity of the virus particle itself. Evidence in support of this possibility comes from studies on the binding of NC, in the context of full-length Gag, to viral genomic RNA. This might concentrate Gag proteins onto one or more RNA molecules, thereby facilitating Gag-Gag multimerization in a template-driven manner. Hence, viral genomic RNA would be a structural element, or scaffold, on which the virion can assemble [116]. Other reports have shown that viral RNA can affect particle morphogenesis [116-119] and structural stability [120,121], although the mechanisms involved are unclear. If RNA structure, or even the dimeric versus monomeric state of the RNA, truly does play a role in virion assembly and/or stability, this might also explain the apparent detection of monomeric RNA in the HIV-1 mutants mentioned above. For example, the duplication of large E/DLS sequences would undoubtedly have altered the overall structure of viral RNA, which might have resulted in the formation of unstable virus particles [102,103]. Degradation of such particles could have indirectly caused the dissociation of dimers that would then appear as monomers on a gel. The fact that these viruses were all noninfectious may also have been due to the formation of unstable virus particles. Consistent with this concept, we found by electron microscopy that HIV-1 mutants lacking DIS stem sequences displayed an increased proportion of immature virus particles [114]. This might mean that either the RNA structure, or the lack of a properly formed dimer, resulted in the production of virus particles with abnormal morphology. Since RNA can affect Gag cleavage, it is possible that mutations in the RNA might have also compromised the cleavage of Gag precursor proteins, which may subsequently have affected particle maturation [122]. We believe that proper RNA dimerization may be a prerequisite for efficient virion assembly and structural stability.

As stated, the link between dimerization and packaging is a subject of ongoing debate [32,33,42,98,99,102,103,109,110], but we and others view dimerization as a prerequisite for packaging. Genomic RNA can be packaged as monomers [99,102,103,105,109], or alternatively as weak dimers that appear as monomers on gels, but mutant viruses that exhibit dimerization defects generally do not grow as well as wild-type viruses. The fact that our ΔDIS-MP2 virus can replicate in tissue culture, despite being severely compromised in genome dimerization, is evidence that efficient dimerization is not required for packaging or replication. In the absence of an authentic DIS, other sequences that affect dimerization may form a weak dimer that allows RNA to be recognized and adequately packaged [87,100,101]. The contribution of the DIS might then be to significantly increase the efficiency of the dimerization process, resulting in more efficient packaging and replication. In conclusion, we agree with opinions expressed by others that the generation of virus particles able to package monomeric genomes is possible, but that dimerization is likely to be a prerequisite for the production of infectious viral progeny [10].

## The DIS as a therapeutic target?

It is clear that virus replication capacity is significantly affected whenever dimerization and/or packaging are compromised, suggesting that these activities can be exploited as anti-HIV drug targets. Indeed, the DIS was first proposed to be a potential therapeutic target at least 10 years ago, and antisense molecules were directed at this region of viral RNA [26,123], without practical outcome. Other approaches directly target the HIV-1 kissing-loop complex, which resembles the eubacterial 16S ribosomal aminoacyl-tRNA site, i.e. the target of aminoglycoside antibiotics such as paramycin and neomycin [124], both of which specifically bind to the kissing-loop complex. Drugs based on antibiotics with high affinity and specificity for the DIS may be a worthwhile approach, although efficacy might be compromised by the fact that HIV can replicate in the face of mutations that decrease genomic dimerization by more than 50% [104].

RNA interference (RNAi) is a novel mechanism that regulates gene expression in which small interfering RNAs direct the targeted degradation of RNA in a sequence-specific manner (reviewed in Lee and Rossi [125]). Although RNAi is a powerful tool, it is not yet clear whether its therapeutic potential will materialize. This not-withstanding, several reports show that specific degradation of HIV-1 RNA is possible in infected cells [125], and reductions of p24 levels by as much as 4 logs have been achieved using RNAi directed against HIV-1 *tat *and *rev *[126]. DNA vectors are currently being engineered that will allow for long-term production of siRNAs for use against chronic diseases, such as HIV-1.

The DIS might also be a good candidate for sequence-specific targeting of HIV by RNAi therapy since it is highly conserved among naturally occurring virus isolates, and, due to its position upstream of the major splice donor, is contained in all HIV-1 RNA transcripts, both spliced and unspliced. Effective DIS-directed degradation of HIV RNA should confer the same viral phenotype as observed with our ΔDIS mutant, which never showed signs of virus replication in either permissive T cell lines or blood mononuclear cells [104]. One concern with use of RNAi is how accessible certain RNA sequences might be. For example, complex secondary structures might cause some sequences to be buried and therefore inaccessible to the siRNA. However, this would not be a concern with DIS-directed RNAi, since the DIS contains a 6 nt palindromic sequence that is believed to initiate the dimerization process by binding to an identical sequence on another molecule of genomic RNA. If two 6 nt stretches of RNA can find each other on two 9200 nt strands of highly structured RNA, they should also be accessible to siRNAs.

Recently, the practicality of RNAi-based therapies against HIV-1 was called into question when it was shown that HIV-1 was able to escape the antiviral pressure of RNAi by generating substitutions or even deletions within RNAi target sequences [127,128]. This again highlights the versatility and plasticity of the HIV-1 genome. However, in these studies, the RNAi target sequences were located within the *tat *and *nef *genes, and the mutations that were generated blocked the effects of the RNAi without conferring any major detriment to virus replication. In contrast, RNAi may be more useful if targeted to more critical RNA elements within the genome, such as the DIS or the Ψ region, since any escape mutations that occur might result in viruses with severely impaired replication ability.

All of these DIS-directed strategies rely on specifically targeting the viral RNA itself, which might not be practical given our inadequate knowledge of the overall structure of the HIV-1 5' region. The fact that RNA sequences such as SL1 and SL3 are known to form relevant RNA-protein interactions raises the possibility that the protein component of these interactions might also provide potential targets for anti-HIV therapy. Such approaches are currently being explored in research aimed at designing inhibitors of the TAR-Tat RNA-protein interaction [129]. Similar approaches might also be developed to target RNA-protein interactions involving SL1 or SL3 and Gag.

## Future directions

Current HIV combination therapies have demonstrated that a multi-targeted approach against the virus results in the greatest degree of suppression of virus replication. Therefore, the identification of novel targets for anti-HIV therapy could significantly improve HIV treatment strategies. HIV-1 RNA dimerization is clearly a critical event that could be exploited as a target once its complete mechanism is elucidated. It is pleasing to see that a number of laboratories that have actively researched RNA dimerization and packaging are now moving beyond conventional *in vitro *and *in vivo *approaches toward more biologically relevant methods. One group has taken chemical modification protocols commonly used for *in vitro *RNA analysis, and adapted them for use in virus-producing cells. Hence, structural analysis of viral RNA, that would previously be carried out only *in vitro *on short fragments of artificially transcribed RNA, can now be performed on *in vivo*-generated HIV-1 genomic RNA (J.-C. Paillart and R. Marquet, personal communication, and [130]). This method also allows comparisons of cellular and virion-derived HIV-1 RNA and represents a middle ground between classic *in vitro *and *in vivo *approaches. The goal of this work is to provide insight on the true structure of the HIV-1 leader, and on which RNA substructures are involved in dimerization. Preliminary data suggest that viral RNA may already be dimerized in the cytoplasm (J-C. Paillart and R. Marquet, unpublished data). This method might also have application in regard to *in vivo *foot-printing that could allow the study of RNA-protein interactions in the context of virus-producing cells.

The structure of the viral RNA that exists in the cell has long been a topic of interest, and recent data suggest that different RNA sequences might be involved in higher order intrastrand structures that favor the dimerization of the two RNA molecules. Such a model has been proposed [131], and is supported by numerous *in vitro *dimerization studies conducted on HIV-1, HIV-2, and SIV RNA [41,131-133]. The model proposes that the HIV-1 5' UTR can form two alternating conformations, termed the long-distance interaction (LDI) and the branched multiple hairpin (BMH) structures. The LDI conformation is believed to exist when the RNA is in a monomer form, and is thought to form a long extended base-paired structure with almost all of the proposed stem-loop sequences buried. This structure is thought to be favored during certain steps of the life cycle, such as translation. In this model, NC has been shown to bind the LDI structure to induce a switch to the BMH structure [131], in which the DIS and ψ would then be exposed in a manner able to mediate dimerization and packaging. Such a 'riboswitch' is an attractive hypothesis, especially since similar mechanisms have recently been proposed to account for previously unexplained results in the field of gene regulation [134]. Although there is currently little *in vivo *evidence directly supporting such a model in the case of retroviruses, the results of previous mutagenesis studies from several laboratories correlate with those that would be predicted from the riboswitch model, both concerning RNA packaging and RNA dimerization status [135]. In regard to dimerization being a prerequisite for packaging, it would also be interesting to test whether an HIV-1 RNA molecule in the LDI conformation can be packaged. Since the BMH conformation is believed to mediate dimerization, one would assume that the LDI structures would not be packageable if dimerization is truly a packaging prerequisite.

Others have developed a fluorescence resonance energy transfer (FRET)-based system to allow visualization of RNA-Gag interactions within cells (A.M. Lever and co-workers, unpublished data). Such a system might provide insight into the timing of genome selection and packaging. It will also be interesting to determine whether this system can be adapted to pinpoint how retroviral RNA dimerization takes place within cells, and whether dimerization indeed occurs before RNA is selected for packaging.

## Competing interests

None declared.

## Author's contributions

RSR gathered the information discussed in this review, and was primary author of the manuscript. CL and MAW carefully read the manuscript and offered insightful suggestions for its revision. All authors read and approved the final version.
